# Spiral aortoplasty for dilated ascending aorta: a new technique for high-risk patients with combined procedures

**DOI:** 10.1186/s13019-019-0960-5

**Published:** 2019-07-18

**Authors:** Rakan I. Nazer

**Affiliations:** Department of Cardiac Science, King Fahad Cardiac Center, School of Medicine, King Saud University, 3642 KSU, Riyadh, 12372-7143 Saudi Arabia

**Keywords:** Aorta, Aortoplasty, Bicuspid valve

## Abstract

Concomitant replacement of the ascending aorta with the aortic valve in patients who have left ventricular dysfunction might carry high operative risks. Performing the conservative reduction aortoplasty was shown to have less complications in such patients. When combined with other concomitant cardiac procedures, the newly described “spiral” aortoplasty technique in this series allows for a mulitplanar wall tension reduction in moderately dilated ascending aorta.

## Introduction

Ascending aorta dilatation is sometimes concomitantly associated with aortic valve disease. It is generally recommended to surgically intervene on the ascending aorta once the maximum diameter is above 4.5 cm in the setting of other surgical cardiac procedures [1]. This is to avoid the possible risk of future aorta dilatation or the possibility of aortic dissection and rupture [2]. The dilated ascending aorta can be surgically addressed by radical ascending aorta replacement with a tube graft or by the less popular and more conservative reduction aortoplasty, with or without external wrapping [3]. The following sections describes a modified reduction aortoplasty with concomitant cardiac procedures in high risk patients.

## Patients

A series of 9 patients are described after local ethical board approval with individual consent for every patient in the series. They had a dilated ascending aortas (4.5 cm to < 5.0 cm) in the setting of concomitant aortic valve replacement with left ventricular dysfunction from July 2016 to December 2018 at our institution. Some patients required additional mitral repair and/or coronary bypass grafting (CABG). All patients successfully underwent “spiral” aortoplasty to reduce the aortic diameter. The ascending aorta was measured for each patient on follow-up at the level of the mid ascending aorta with the use of CT angiography (Table 1).

**Table 1:** Case series of 9 patients who underwent “spiral” aortoplasty with concomitant aortic valve replacement +/− CABG +/− mitral valve repair

Series no.	Age / Sex	AAD before surgery	LV EF	Diagnosis	Bicuspid aortic valve	Euro Score	Procedure	Follow-up time	Condition on follow-up
1	53 M	4.7 cm	30%	CHF	+	54.50%	Aortoplasty	30 months	Stable. AAD 3.8 cm
				Severe AI			AVR (mosaic #27)		
				Moderate MR			MV repair (annuloplasty #30)		
				Atrial fibrillation			MAZE		
				Stroke			LAA clot extraction and ligation		
				Clot in the LAA					
2	70 M	4.5 cm	25%	CHF	+	20.40%	Aortoplasty	28 months	Stable. AAD 3.6 cm
				Severe AI			AVR (mosaic #27)		
3	60 M	4.5 cm	35%	Severe AS		17.70%	Aortoplasty	25 months	Stable. AAD 3.7 cm
				CAD: LAD stenosis			AVR (mosaic #25)		
							CABG: LIMA-LAD		
4	67 M	4.6 cm	30%	Severe AS		20.20%	Aortoplasty	21 months	Stable. AAD 3.8 cm
				Moderate MR			AVR (mosaic #21)		
				CAD: LAD stenosis			MV repair (annuloplasty #28)		
							CABG: LIMA-LAD		
5	62 F	4.5 cm	40%	Severe AS	+	9.50%	Aortoplasty	17 months	Stable. AAD 3.3 cm
				Mild MR			AVR (mosaic #23)		
				CAD: ostial RCA stenosis			CABG: SVG-RCA		
6	55 M	4.8 cm	15%	Severe AI	+	24.00%	Aortoplasty	16 months	Stable. AAD 3.9 cm
				CAD: 3 vessel disease			AVR (mosaic #27)		
							CABG: LIMA-LAD, SVG-OM, SVG-PDA		
7	50 M	4.8 cm	20%	CHF	+	84.70%	Aortoplasty	14 months	AAD 3.7 cm. The patient presented with aortic prosthesis endocarditis and aortic root abscess 10 months after the first operation. He underwent redo surgery with aortic annulus reconstruction and AVR. The patient was discharged on chronic dialysis and died 4 months later due to refractory heart failure.
				Severe AI			AVR (mosaic #27)		
				Severe MR			MV repair (annuloplasty #30)		
				Chronic kidney disease (creatinine 200 μmol/L)					
8	66 M	4.6 cm	40%	Severe AI		12.80%	Aortoplasty	6 months	Stable. AAD 3.5 cm
				CAD: RCA total occlusion			AVR (mosaic #25)		
							CABG: SVG-PDA		
9	72 M	4.5 cm	40%	Severe AS	+	11.00%	Aortoplasty		
				CAD: 3 vessel disease			AVR (mosaic #23)	4 months	Stable. AAD 3.5 cm
							CABG: LIMA-LAD, SVG-OM, SVG-PDA		

*AA* ascending aorta, *AAD* ascending aorta diameter, *AI* aortic regurgitation, *AS* aortic stenosis, *AVR* aortic valve replacement, *CABG* coronary artery bypass grafting, *CAD* coronary artery disease, *CHF* congestive heart failure, *LAA* left atrial appendage, *LAD* left anterior descending, *LIMA* left internal mammary, *LV EF* left ventricular ejection fraction, *MR* mitral regurgitation, *OM* obtuse marginal, RCA: right coronary artery, *PDA* posterior descending artery, *SVG* saphenous vein graft

## Technique

**Fig. 1:**
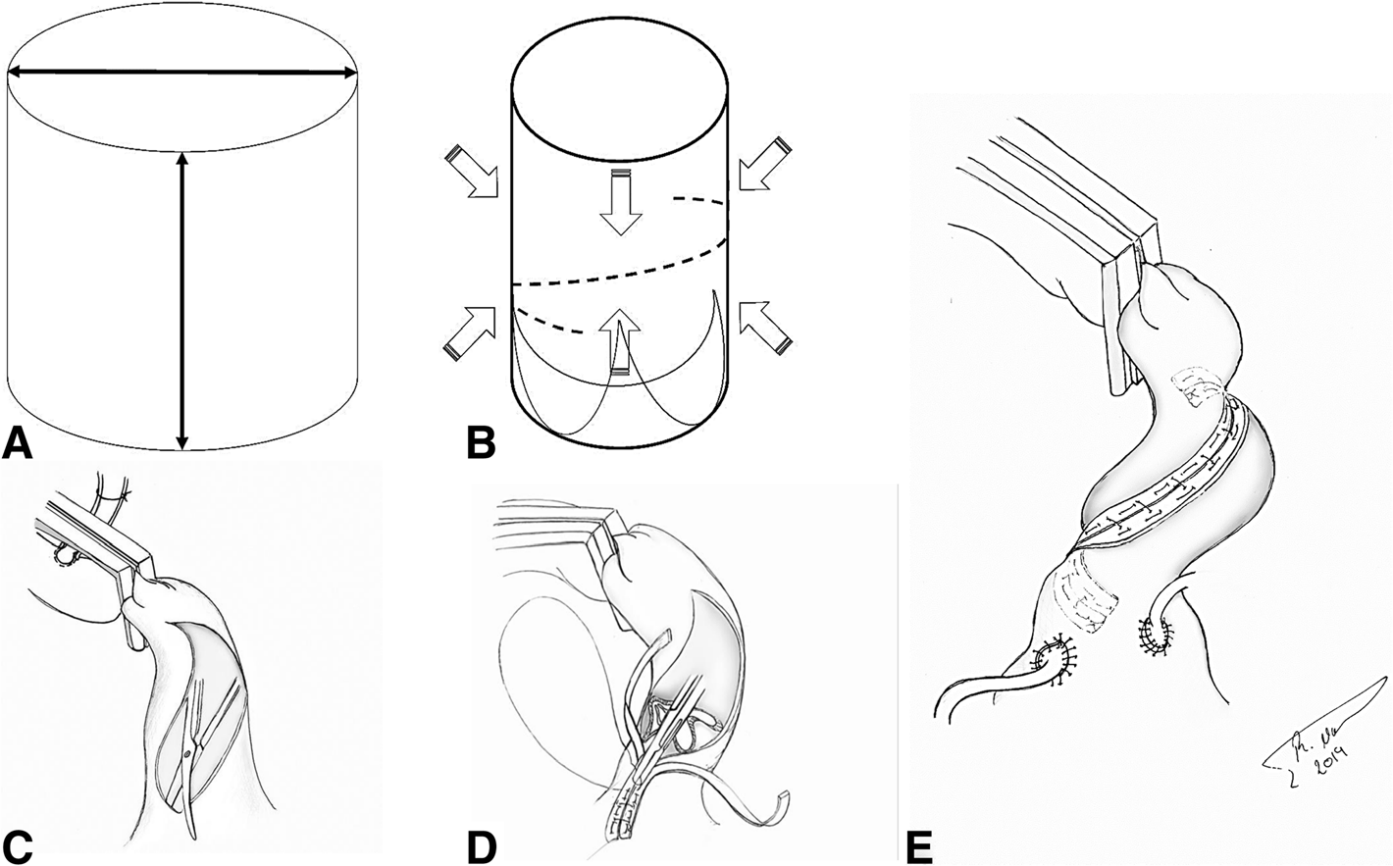
**a** The expansion of the aorta occurs on a multiplanar axis. **b** “Spiral” aortoplasty allows for the circumferential wall tension reduction along multiple planes. **c** The aortotomy is performed in a spiral fashion extending from the pulmonary bifurcation and down to the annulus of the non-coronary sinus. **d** The aortotomy is sandwiched between two strips of Teflon felt incorporating 0.5 cm of tissue on either side. **e** The proximal coronary grafts are implanted either above or below the suture line

The heart was accessed through a median sternotomy and cardiopulmonary bypass was initiated via central cannulation. The arterial cannula was inserted in the proximal aortic arch. Prior to applying the cross clamp, an umbilical tape was looped around the backside of the ascending aorta and was lifted before applying the cross clamp to ensure the clamp captured the entire aortic wall. The clamp was positioned as high as possible in the ascending aorta. An oblique aortotomy was performed in the middle of the anterior segment of the ascending aorta and was spirally extended downward and laterally toward the non-coronary sinus, stopping at the aortic annulus, and superiorly toward the left shoulder up to the bifurcation of the pulmonary artery. The aortic valve was replaced though this access. Concomitant procedures such as distal coronary grafts and/or mitral repair were performed prior to the aortic procedure. The aortotomy closure was sandwiched between 2 strips of Teflon felt and sutured together using a double closure technique. The Suture incorporated 0.5 cm of aortic tissue on either side during closure in order to reduce the aortic size by 1 cm. (Fig. 1).

## Comments

Fusiform ascending aorta aneurysm is commonly observed in patients with aortic valve disease, particularly in those with a bicuspid aortic valve. The dilatation is likely due to intrinsic factors harbored within the connective tissue of the aortic wall. The multiplanar dilatation causes elongation and circumferential expansion of the aorta. Previously described reduction aortoplasty techniques primarily reduce the aortic diameter and wall tension at a single plane in the mid-anterior wall of the ascending aorta [4]. The technique described in this series has the same advantages of other described aortoplasty approaches in high-risk patients with the added advantage of reducing the wall tension and size along the circumferential and longitudinal planes. External wrapping was avoided because of the reported risks of “under-the-wrap” aortic atrophy and rupture, wrap migration, and the need to construct proximal coronary anastomosis in the ascending aorta. Bicuspid aortic valve was present in over half of the series. All patients showed an approximately 1-cm reduction of the mid-ascending aortic diameter on echocardiographic and tomographic follow-up. This technique should not be used in patients with dilated aortic root, ascending aortas with a diameter of > 5.0 cm, in patients with acute aortic dissection, or in patients with syndromic connective tissue disorders such as Marfan’s syndrome. The series is limited by its small size, short follow-up, and the potential risk for re-dilatation of the ascending aorta.

In summary, this series describes a limited number of high risk patients who successfully underwent concomitant aortic valve replacement with reduction aortoplasty and other added procedures. The spiral aortoplasty technique has the advantage of multiplanar aortic size reduction without the need to perform the more radical ascending aorta replacement in moderately dilated ascending aorta.

## Data Availability

Data related to the manuscript are available upon request.

## Abbreviations

AA: Ascending aorta
AAD: Ascending aorta diameter
AI: Aortic regurgitation
AS: Aortic stenosis
AVR: Aortic valve replacement
CABG: Coronary artery bypass grafting
CAD: Coronary artery disease
CHF: Congestive heart failure
LAA: Left atrial appendage
LAD: Left anterior descending
LIMA: Left internal mammary
LV EF: Left ventricular ejection fraction
MR: Mitral regurgitation
OM: Obtuse marginal
PDA: Posterior descending artery
RCA: Right coronary artery
SVG: Saphenous vein graft
